# Chitogel improves long-term health economic outcomes following endoscopic sinus surgery in severe chronic rhinosinusitis patients

**DOI:** 10.3389/frhs.2024.1196499

**Published:** 2024-02-28

**Authors:** Jacqueline M. Barber, George Bouras, Grace S. Robinson, Simon R. Robinson

**Affiliations:** ^1^ Chitogel Limited, Wellington, New Zealand; ^2^ Department of Surgery – Otolaryngology Head, and Neck Surgery, Central Adelaide Local Health Network, Adelaide, SA, Australia; ^3^ Wakefield Hospital, Wellington, New Zealand

**Keywords:** chronic rhinosinusitis, Chitogel, microbiome, endoscopic sinus surgery, health economic outcomes

## Abstract

**Introduction:**

Chronic rhinosinusitis causes severe symptoms that can affect patient quality of life. Endoscopic sinus surgery can be effective in improving symptoms, although surgical outcomes can be compromised post-operatively, and revision surgery is required in a proportion of patients. This study compares outcomes and healthcare resource use in patients undergoing sinus surgery with or without Chitogel as a post-operative dressing.

**Methods:**

A retrospective cohort study was conducted using deidentified audit data from adult patients with severe chronic rhinosinusitis, who underwent endoscopic sinus surgery between January 2016 and December 2021. Patients in the intervention group received Chitogel as a post-operative dressing, and control patients received standard best-practice care. Cox Proportional Hazards survival analysis was used to compare revision surgery rates and time to revision between treatment groups. The rate of revision surgery was used to estimate potential health sector savings associated with use of Chitogel following surgery compared to the control arm, considering initial treatment costs and the cost of revision surgery.

**Results:**

Over 18–24 months, patients treated with Chitogel demonstrated significantly lower rates of revision surgery (*p* = 0.035), and a trend towards decreased use of post-operative steroids, compared to control. Potential health sector savings due to reduced rates of revision surgery following use of Chitogel are estimated as NZ $753,000 per 100 patients.

**Conclusion:**

Severe chronic rhinosinusitis patients treated with Chitogel had lower rates of revision surgery within the first 18–24 months post-operative. These findings suggest that use of Chitogel can improve long-term patient outcomes and should improve health system efficiency.

## 1 Introduction

Chronic rhinosinusitis (CRS) is an inflammatory condition affecting the paranasal sinus mucosa and nasal passages for 12 weeks or longer causing two or more of the following symptoms: nasal congestion, mucus discharge, facial pain or pressure, loss of smell. CRS affects 2%–16% of the US population (1–3), with estimated direct healthcare costs ranging between US$7 billion and US$13 billion (4, 5), and an additional US$13 billion associated with indirect healthcare costs (4). These costs are worn by the patient, employers, third-party insurance companies, taxpayers and wider society, although the proportions differ depending on the specific economic market. It is, therefore, critically important to investigate care options available to clinicians to ensure their delivery of cost-effective care.

Successful management of CRS often requires multiple concurrent therapies. Current primary care treatment options for CRS include symptom management through use of corticosteroids, antibiotics, saline irrigation, and leukotriene receptor agonists (6, 7). In an estimated 60% of cases, endoscopic sinus surgery (ESS) is utilised as an adjunct to medical therapies (8). Initial ESS is not curative in all cases, and post-operative outcomes can be compromised by factors including middle turbinate lateralization, incomplete anterior or posterior ethimoidectomy, frontal recess scarring, middle meatal antrostomy stenosis (9). Adhesion (scar) formation and ostial stenosis are common causes of surgical failure (10). Avoiding middle turbinate destabilization during surgery may also improve the success rate of primary ESS (9, 11). Long-term ESS success is also affected by parameters including severity and type of disease (12), patient history, co-morbidities (13–15), ethnicity (16) and other risk factors (17). Nevertheless, ESS is still considered to be cost-effective in intermediate and long-term disease management (18).

A clear dependence between revision rates and a patient's initial disease state, particularly the presence of nasal polyps has been widely identified (19–22), although the impact of other respiratory conditions such as asthma and cystic fibrosis on revision surgery rates has also received attention (14, 15). Philpott et al. found 57% of patients presenting with CRS with nasal polyps (CRSwNP) and allergic fungal rhinosinusitis had undergone previous endoscopic nasal polypectomy, and 46% had required more than one operation (21). In contrast, surgical rates in CRS patients without nasal polyps (CRSsNP) were significantly lower; 13% of cases specifically reported ESS, and of those only 30% reported multiple procedures (χ^2^ *p* < 0.001). This corroborated results from an earlier audit of sinonasal patient outcomes in the UK (20). In line with this, a longer-term cohort study investigated surgical outcomes in CRSwNP patients, finding that 78.9% of patients with CRSwNP experienced disease recurrence and 36.8% underwent revision surgery over a 12-year period (19). Similarly, Bayer et al. recently reported a 78.9% rate of revision surgery in CRSwNP patients compared with 21.1% in CRSsNP patients over a three-year period (23).

Disease severity therefore directly impacts per patient costs. Over one year, a 27% increase in costs was reported for US CRSwNP patients compared to CRSsNP patients, largely attributed to revision procedures required during the period (24, 25).

The sinus microbiome also influences long-term sinus health and the need for revision surgery (26). Commensal bacteria are well-understood to inhibit the growth of pathogenic bacteria through their secretion of bacteriocins that are exclusively inhibitory to pathogenic bacteria (27). *Cutibacterium acnes* is renowned for this (28), and has shown antimicrobial activity against *Staphylococcus aureus* and methicillin-resistant *S. aureus* pathogens isolated from the sinuses of chronic rhinosinusitis patients (29). Commensal corynebacteria, such as *Corynebacterium accolens* are also associated with healthy sinuses and are known to inhibit the growth of pathogenic species (30). Consistent with this, Paramasivan et al. detected a significant reduction in relative abundance of Corynebacterium among patients suffering from CRS with nasal polyps (31). Therefore, combining surgical interventions with treatments that support ongoing sinus health could have significant benefits on long-term patient outcomes (26).

The Chitogel Endoscopic Sinus Surgery Kit (Chitogel, manufactured by Chitogel Limited) is a biodegradable post-operative dressing that was cleared by the US Food and Drug Administration in 2017. Chitogel is a viscous and pliable gel made from two biodegradable polymers and is applied to the dissected cavities of the frontal, maxillary, ethmoid and sphenoid sinuses after surgery. Chitogel conforms to the cavities and creates a physical barrier, supporting and separating tissues compromised by the surgery to prevent adhesion formation and minimise ostial stenosis. It controls minimal bleeding by stimulating platelet aggregation but has no effect on the clotting cascade, reducing the body's inflammatory response, and aiding natural healing (32, 33).

Chitogel has demonstrated significant clinical benefits, resulting in quantified improvement in all sinus ostia sizes and positive effects on wound healing (10, 34). It has been shown that the frontal ostia of patients treated with Chitogel were 64% of the original size, almost double the size of the control ostia (37%) at 12 months follow-up (10). Compared with the control group, the same patients experienced significant reductions in adhesions, oedema, granulations, crusting and infection, and improved long-term ostial patency (10). In a recent meta-analysis of chitosan-based dressings used for the prevention of adhesions and improvement in wound healing, Chitogel was the dressing used for most of the studies and was shown to significantly reduce adhesions and improve haemostasis compared to controls (35). Beneficial effects of Chitogel on the sinus microbiome of CRS patients undergoing ESS have also been identified (26). The combined relative abundance of commensal bacteria *Corynebacterium* sp. and *Cutibacterium* sp. was significantly increased from 30.15% to 46.62% at 12 weeks after post-operative treatment with Chitogel compared to control (47.18%–40.79%). Treatment with Chitogel resulted in improved endoscopic appearance of the sinuses (*p* = 0.03) and ostial patency were noted after treatment with Chitogel compared with control at 12 weeks (*p* < 0.001) (26).

Escalating healthcare costs world-wide make it important to understand the true costs of care options, for example whether adopting a new technology is cost-effective and to the benefit of patients. Direct and indirect costs associated with a given treatment pathway should both be considered. A recent study in a public Australian Hospital found that use of Chitogel following ESS resulted in fewer follow-up consults per patient (5.4) in comparison to control (6.8), with obvious fiscal impacts (36). Given the clear patient benefits delivered by Chitogel, healthcare providers and third-party payers must consider the potential direct and indirect financial benefits of introducing Chitogel into standard post-operative care for ESS. This study aims to establish the long-term financial impact of Chitogel when used as a post-operative dressing for ESS from the perspective of a private New Zealand hospital. Chitogel is expected to reduce the need for post-operative interventions such as revision surgeries and affect use of antibiotics and corticosteroids, by supporting healthy sinuses, and preventing adhesions and stenosis to improve long-term ostial patency.

## 2 Materials and methods

### 2.1 Clinical data source

Deidentified patient data from a single fellowship-trained rhinologist, Mr Simon Robinson (SRR, Wakefield Hospital, Wellington, New Zealand) was used. The dataset contains certain demographic descriptors (age at surgery and sex), details of diagnosis and pre-operative clinical measurements (eosinophil count), post-operative CRS treatment details, and complies with the Health Information Privacy Code (New Zealand, 2020). The study was notified to the New Zealand Health and Disability Ethics Commission, although review was not required.

### 2.2 Study design

A retrospective cohort study was conducted using deidentified data from adult patients aged 18 years or over with severe CRS who underwent bilateral full-house functional endoscopic sinus surgery between January 2016 and June 2021. Disease severity was established using the Lund-Mackay (LM) (37) scoring system of peri-operative computerized tomography (CT)-scans and defined as a score of 15 or greater. Patients who had had previous surgical treatment for sinuses, and who attended no post-operative follow-up appointments were excluded.

### 2.3 Patient selection and cohort assignment

Chitogel was introduced into SRR's practice as standard post-operative care for ESS on 8 April 2019. All severe CRS patients undergoing ESS on or after this date, who met the inclusion criteria, attended initial follow-up appointments, and whose initial surgery was at least 18-months prior to the date of analysis (21 December 2021) were included in the intervention group. Active controls were patients that received surgery between 1 January 2016 and 7 April 2019. Patients were followed for up to 24 months.

Patient selection was based on pre-operative CT scans and staging using the LM scale to measure disease severity, which was consistent with current literature (38–41). Pre-operative eosinophil counts were taken to measure disease-state balance between treatment groups. Sex and age at the time of surgery was recorded, although patients were not matched based on demographic or clinical parameters.

### 2.4 Outcome measures

Over the follow-up period following ESS, revision surgery, and uptake of rescue medications including oral or topical corticosteroid or antibiotics were monitored. Rescue medications were defined as those received more than 1-month post-surgery. All patients received a standard two-week post-operative course of antibiotics and steroid rinses, which were excluded from data collection and analysis.

### 2.5 Statistical modelling

#### 2.5.1 Demographics and pre-operative measurements

Statistical modelling and analysis were conducted with R v 4.2.0 (42). Significance was assessed at a 95% level of confidence (*p* = 0.05). Pre-operative LM scores and eosinophil counts and age at surgery were compared between Chitogel treated and control groups using Student's *t*-test. Fisher's Exact test was used to compare differences in sex between groups.

#### 2.5.2 Time to revision

Cox Proportional Hazards survival analysis models were used to model whether there was a significant association between revision surgery, time since the initial surgery, and treatment group. Specifically, the “coxph” function from the “survival” R package was used. No covariates were included in these models other than the treatment group, yielding a hazard function h(t) in the form of Equation 1 below:

(1) $$h(t)=h_{o}(t)\;\times \exp(b*\mathrm{group})$$

where exp(b) is the hazard ratio representing the hazard ratio of requiring revision surgery in the Control group (coded as group = 1) as opposed to the Chitogel group (coded as group = 0). In these models, right censoring was used, with, patients undergoing revision surgery coded as “1” while all other patients were coded as “0” for the length of their follow-up time or the time cutoff, whichever was shorter. 1- and 2-year follow-up analyses were conducted.

Patients whose revision surgery occurred after the cutoff were right-censored, e.g., for the purpose of the 1-year follow-up analysis, a patient who had revision surgery at 500 days was considered as not having had revision surgery. Significance was assessed at a 95% level of confidence (*p* = 0.05). 1- and 2-year survival models were conducted.

#### 2.5.3 Revision rate over the study period

All hazard ratio and confidence interval calculations were based on Cox Proportional Hazards models. All confidence intervals were calculated at a confidence level of 95%. All hazard ratios can be interpreted per the following example: a hazards ratio of 2.39 in the 2-year model means that over 2 years post-surgery a patient in the control group is 2.39 times more likely to require a revision surgery than a patient treated with Chitogel. All estimations of revision rates over a certain period were calculated as simple percentages.

#### 2.5.4 Antibiotic and steroid uptake

Patient notes were interrogated using text matching to determine post-operative use of steroids or antibiotics in addition to standard care. A patient was determined to have received a steroid course if prednisolone, budesonide, or a general note indicating steroid use appeared in their notes 1 month or more post-surgery. Similarly, a patient was determined to have received antibiotics if mupirocin, ciprofloxacin, augmentin, azithromycin, roxithromycin or a general comment indicating antibiotic usage appeared in the notes 1 month or more post-surgery. As steroid and antibiotic course lengths could not be quantified due to data limitations, binary encoding was used: “0” indicating no steroid/antibiotic use and “1” indicating that patient had received steroid/antibiotics more than 1-month post-surgery. Fisher's Exact tests were used to compare post-operative steroid and antibiotic usage between groups.

### 2.6 Cost data

Costs estimates are from the perspective of the party responsible for payment, e.g., the patient or insurance provider. Estimated unit costs for bilateral function ESS with frontal recess dissection and external septoplasty, endoscopic modified Lothrop revision surgery (EML) were calculated using price estimates from the audit facility, Wakefield Hospital (Wellington, New Zealand) in 2022. Although post-operative follow-up occurred over multiple years in some cases, for simplicity all estimates are based on the 2022 costings. These include overnight accommodation charges; operating theatre and recovery room fees on a per minute occupancy; medical supplies including the Chitogel device, peri-operative medication such as corticosteroid, post-operative irrigation solutions, surgical consumables, and anaesthetic; and professional fees of the surgeon and anaesthetist. The cost of Chitogel was calculated assuming a NZ$350 selling price per single device, which is sufficient for post-operative use in a single patient after all standard ESS approaches. Post-operative medications listed in Section 2.5.4 are publicly funded medications in New Zealand, so are not included in the estimates.

## 3 Results

### 3.1 Demographics

A total of 59 patients met all inclusion criteria, 30 in the Chitogel treated group and 29 in the control group (see Supplementary Information). There were no significant differences in age (*p* = 0.2), pre-operative eosinophil count (*p* = 0.64) or pre-operative LM scores (*p* = 0.97) between treatment groups. However, there were more male patients in the Chitogel treated group—21/30 patients in the Chitogel group were male, while only 13/29 in the control group were male (*p* = 0.07) (Table 1).

**Table 1:** Demographic summary tables.

	Control (*n* = 29)	Chitogel (*n* = 30)
Sex		
Female	16 [55%]	9 [30%]
Male	13 [45%]	21 [70%]
Age	51.9 (12.6)	47.3 (14.8)
LM	17.7 (2.36)	17.6 (2.59)
Pre-operative eosinophil count	0.4 (0.351)	0.361 (0.191)

Each outcome measure is expressed as mean [%] (sd).

### 3.2 Time to revision surgery rate analysis

In the control group, 18/29 (62.1%) of patients required revision surgery within 2 years, and 13/29 (44.8%) required revision within 1 year. In the Chitogel treated group, only 9/30 (30.0%) of patients required surgery within 2 years, and 7/30 (23.3%) required revision within one year.

**Figure 1:**
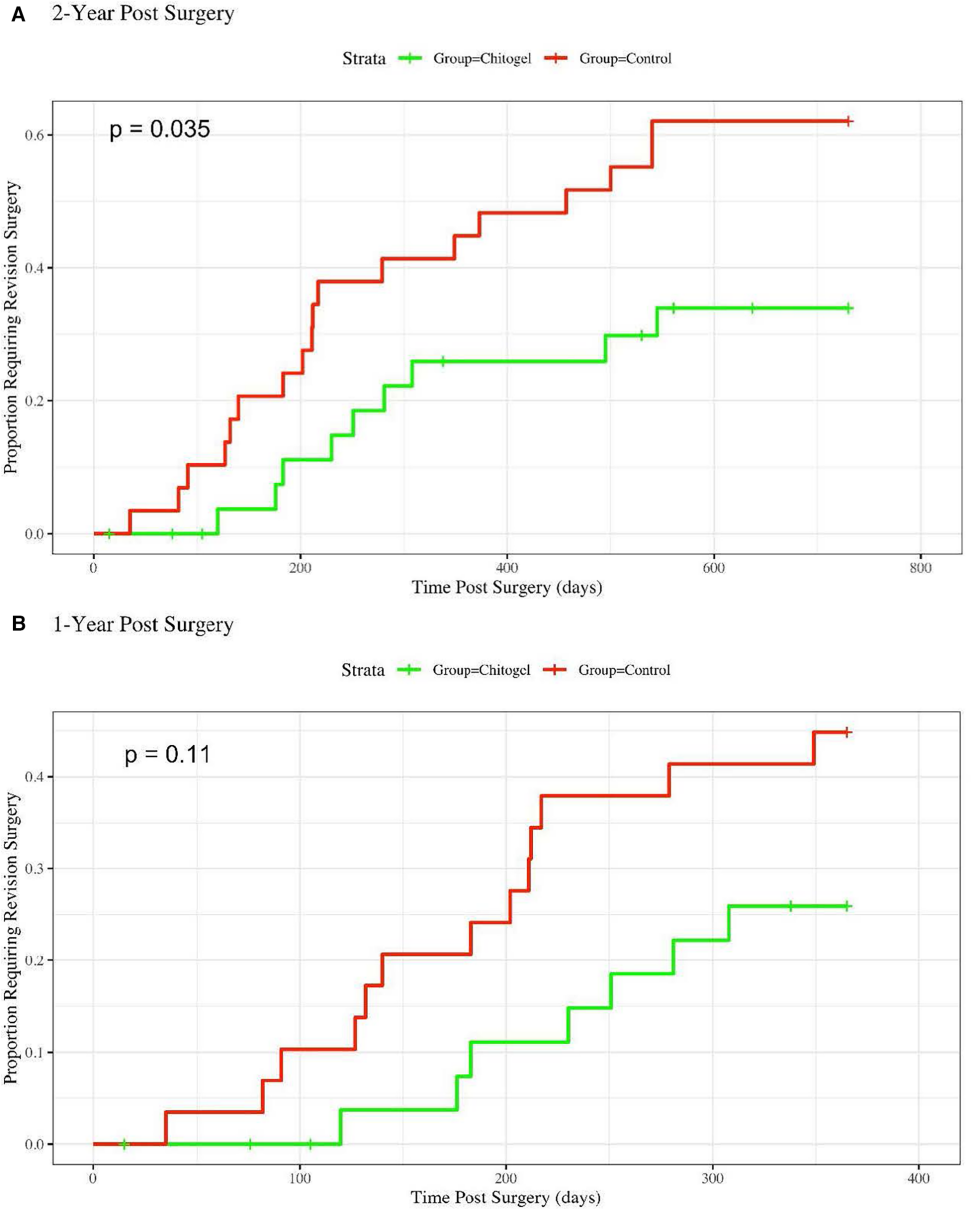
Hazard rate plot showing the proportion of patients requiring revision surgery over: (**A**) 2-years post-surgery, hazards ratio = 2.39 (1.12, 5.077), *p* = 0.035*; and (**B**) 1-year post-surgery, hazards ratio = 2.1 (0.84, 5.27), *p* = 0.11.

There was a significant reduction in the revision surgery rates within 2 years post operation based on the survival analysis (*p* = 0.035) (Figure 1A). The 1-year analysis shows the same trend, although it is not statistically significant (*p* = 0.11). The divergence in revision surgery rates begins within the first 100 days, where the Kaplan–Meier curves begin to diverge (Figure 1B).

For 2-year revision surgery analysis, the Cox Proportional Hazards model coefficient was 0.87, yielding a hazards ratio of 2.39 with a 95% confidence interval of (1.12, 5.077). Accordingly, over 2 years post-surgery, a patient in the control group was on overage 2.39 times more likely to require a revision surgery than a patient treated with Chitogel. For the one 1-year analysis, the hazards ratio was 2.10 (0.84, 5.27), indicating that a patient in the control group was on average 2.10 times more likely to require a revision surgery than a patient treated with Chitogel.

Despite differences in revision surgery rates between groups, the average time between initial and subsequent surgeries is similar between control and intervention arms: 41.0 weeks for control (sd 23.22), 36.9 weeks for Chitogel (sd 20.5 weeks). The proportion of total surgeries that occurred in both groups within the first year is also similar: 13/18 (72.2%) surgeries in the control group, compared with 7/9 (77.7%) in the Chitogel-treated group.

### 3.3 Antibiotic and steroid usage

There were no statistically significant differences in steroid or antibiotic usage between groups more than 1-month post-surgery. For steroids, 6/30 (20%) of Chitogel patients were prescribed steroids post-surgery, compared to 10/29 (34.5%) of Control patients (*p* = 0.25). For antibiotics, 6/30 (20%) of Chitogel patients were prescribed antibiotics post-surgery, compared to 7/29 (24.1%) of Control patients (*p* = 0.76).

### 3.4 Societal and health sector savings

**Figure 2:**
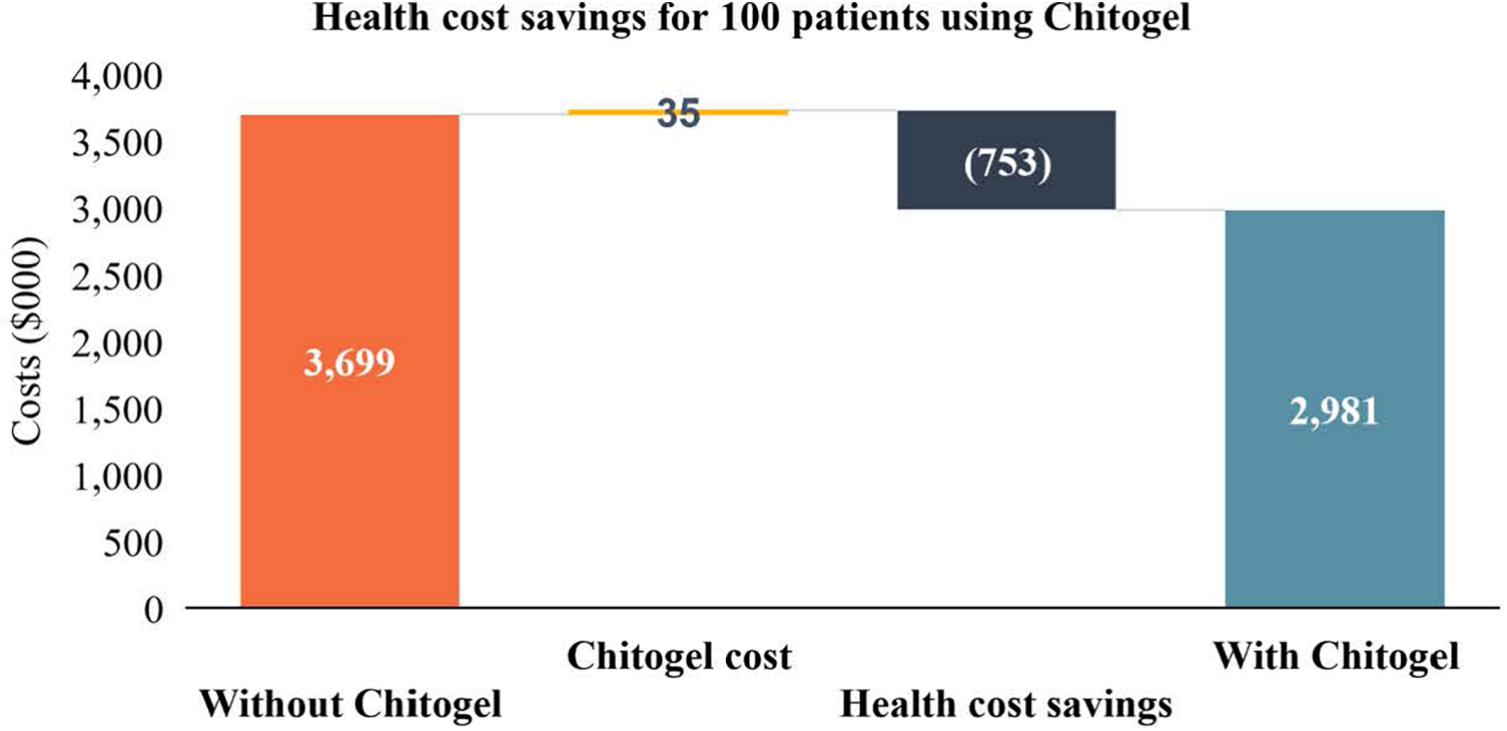
Health cost savings for 100 patients using Chitogel. The direct health system cost savings through use of Chitogel can be estimated for a cohort of 100 assuming the 2.39-fold reduced rate of revision surgery for patients treated with Chitogel compared with control, initials surgery cost for the cohort ($22,421.01 × 100 = $2,242,101.00), cost of Chitogel for the cohort ($35,00), and revision surgery cost for the cohort ($23,454.01 × 32 = $750,528.32).

Potential health sector savings due to initial use of Chitogel in a cohort of 100 patients are estimated as NZ$753,000 (Figure 2). These are calculated using the rate of revision surgery following use of Chitogel after initial ESS in comparison to the control arm, subtracting the initial cost of the product and surgery, and adding the estimated cost of EML, a common revision surgery (Table 2).

**Table 2:** Estimated hospital charges and professional fees for standard ESS involving bilateral functional ESS frontal recess dissection and external septoplasty, and EML revision surgery.

	Standard ESS—bilateral functional ESS frontal recess dissection and external septoplasty				Revision surgery—EML			
	Estimate		Price, 2022 ($NZ)		Estimate		Price, 2022 ($NZ)	
	Min	Max	Min	Max	Min	Max	Min	Max
Hospital charges								
Accommodation, private room (per night)	1	2	865.22	1,730.44	1	2	865.22	1,730.44
Operating theatre (minutes)	75	90	2,460.87	2,926.09	75	90	2,460.87	2,926.09
Recovery room (minutes)	60	75	292.17	365.22	40	60	194.78	292.17
Resident Medical officer per (night)	1	2	73.91	147.83	1	2	73.91	147.83
Medical supplies			3,500.00	4,000.00			6,000.00	6,500.00
Image guidance			NA	NA			1,000.00	1,200.00
Miscellaneous analyses (eg ECG)			300.00	400.00			300.00	400.00
*Total hospital charges (excl GST)*	* *		*7,492.18*	*9,569.57*	* *	* *	*10,894.79*	*13,196.53*
*Total hospital charges (incl 15% GST)*	* *		*8,616.01*	*11,005.01*	* *		*12,529.01*	*15,176.01*
Professional fees								
Surgeon's fee	* *		10,200.00	10,200.00	* *		8,000.00	8,000.00
Anaesthetist's fee	* *		1,500.00	1,800.00	* *		1,500.00	1,800.00
*Total professional fees (excl GST)*	* *		*11,700.00*	*12,000.00*	* *		*9,500.00*	*9,800.00*
*Total professional fees (incl 15% GST)*	* *		*13,455.00*	*13,800.00*	* *		*10,925.00*	*11,270.00*
Treatment								
Chitogel	* *		350.00	350.00	* *		NA	NA
***Total cost (incl 15% GST)***	*** ***		***22,421.01***	***25,155.01***	*** ***		***23,454.01***	***26,446.01***

Indirect cost burdens associated with revision surgeries and other subsequent treatment include surgeon and theatre time associated with revision surgery (75 min), and the cost of patient days off work associated with surgery. Costs associated with missed working days were calculated using the estimated total number of missed working days per patient (10) multiplied by the estimated median daily wage (NZ, 2021, Statistics NZ, NZ$29.66/h = NZ$237.28/day). The total time and financial cost have been extrapolated to a cohort of 100. Assuming the 2.39-fold reduced rate of revision surgery for patients treated with Chitogel compared with control (Figure 2), 32 revision surgeries can be avoided in a cohort of 100 patients, avoiding 320 working days off and saving NZ$ 75,929.60 in lost wages. Conservatively assuming 75 min per revision surgery, 40 h of theatre time is also saved, in addition to professional surgeon and anaesthetist costs (Table 2).

## 4 Discussion

### 4.1 Post-operative outcomes

This retrospective observational study demonstrated reduction in revision surgeries conducted during the 2-year patient follow-up period among severe CRS patients (LM ≥15) who underwent FESS with Chitogel used as a post-operative dressing, vs. those who underwent FESS alone. Only 30.0% of patients treated with Chitogel required revision surgery over the 2-year period compared with 62.9% of patients in the control group, a statistically significant difference (*p* = 0.035). Consistent with this, the Cox Proportional Hazards Ratios show that a patient in the control group was 2.10 and 2.39 times more likely to require a revision surgery than a patient treated with Chitogel at 1-year and 2-year post-surgery timepoints, respectively. Differences between groups became evident within the first 100 days post-surgery. Uptake of post-operative medications prescribed more than 1-month post-surgery was analysed, although no differences were observed between groups for either class of medication.

Revision surgery rates reported here are consistent with studies that focus on severe CRS patients (LM score ≥15) (19, 23, 38), although the revision surgery rates for this severe CRS cohort are higher than some reports that do not differentiate between initial disease severity. Naidoo et al. investigated long-term frontal ostium patency rate and symptom improvement, and the need for further revision surgeries in severe CRS patients undergoing primary ESS and EML. The study showed that risk of requiring EML following previous surgery was 45%, which increased significantly to 67%–75% in patients with a preoperative LM score of >16, compared to 7.6%–22% in patients with non-severe preoperative LM scores (38).

In this current study, the average time between initial and subsequent surgeries is similar between control and intervention arms: 41.0 weeks for control and 36.9 weeks for Chitogel, indicating that the study observation period is sufficient to capture a large proportion of revision surgeries required in both arms. Some variation in average times between initial and subsequent surgeries time is reported in the literature, although such figures are generally for cohorts that include all levels of disease severity. For example, a US-based population study over a 10-year period showed the average time between the initial and subsequent surgeries was 4.39 years, with an overall revision surgery rate greater than 15% (17).

Post-operative outcomes are restricted to patients operated upon by a single rhinologist at a single center. Nevertheless, the general outcome trends can be applied to other practices in the public and private sector within New Zealand and other markets, and suggest that Chitogel following ESS procedures may have a long-term positive impact on patients.

### 4.2 Health economics modelling

Cost impact analysis of innovative healthcare technologies is increasingly utilised by third-party payers and healthcare providers, particularly those in the public sector, as a tool to guide decision making around whether to adopt a given technology (43). Revision surgery rates reported here can be used in conjunction with cost estimates for the product and surgery (initial and revision) (Figure 2, Table 2) to estimate costs for larger cohorts and impact to the wider health system. The current cost structures are specific to one private practice in the New Zealand market, although the principles can be applied to various scenarios in the public and private sector within New Zealand and other markets and can be used to assess the fiscal and societal impacts of Chitogel.

From the overall revision surgery rates, one can calculate that use of Chitogel during initial ESS in a cohort of 100 patients could avoid 32 revision surgeries increasing health system efficiency. When multiplied by the minimum financial and time costs of revision surgery this leads to a total health system saving of NZ$753,000.00, and at least 40 h of theatre and surgical time taking into consideration the minimum unit cost of initial surgery and initial cost of Chitogel (Figure 2). An estimated NZ$75,929.60 is also associated with the 320 days off that can be avoided through initial use of the product within the cohort assuming 10 days off per patient. These are conservative estimates based on the minimum time and cost requirements of standard revision surgeries. Cost and health system efficiency savings vary if one considers longer surgical times (Table 2), while impact to work-place productivity depends on the leave taken by each patient.

Given these patient benefits and conservative cost-savings, Chitogel can be viewed as an investment, with the long-term cost-savings and improvements to patient welfare far outweighing the initial cost of the product. This is consistent with other studies that have found ESS to be the most cost-effective intervention within the third year after surgery in comparison to continued medical treatment both fiscally and with regards to quality of life (5). A similar trend to reduced health system costs and increased efficiencies following use of Chitogel has been observed in an Australian public hospital through a clear reduction in the need for follow-up consults (36).

### 4.3 Limitations

There are inherent limitations to retrospective study designs and use of their data, which are addressed below.

Patients included in the study received treatment from a single rhinologist at a single center.

The retrospective nature of the clinical data and the size of the cohort did not allow for patient propensity matching for demographic characteristics (sex, ethnicity), relevant conditions, or comorbidities. The study groups were balanced with regards to pre-operative disease state based on LM scores and eosinophil counts, and age.

There were more males receiving Chitogel than females (21/30, 70% male) compared to the control cohort (13/29, 45% male). Although the overall cohort examined in this study was relatively balanced with regards to sex, 25:34 female to male (42%).

Patients who had previously undergone sinus surgery were excluded, as such patients may have been pre-disposed to requiring revision.

The study was not blinded: patients and the surgeon were aware of the post-operative dressing received. Regardless, patients in both treatment arms received post-operative care that was considered standard best practice at the time of surgery.

Data for each patient was collected directly from surgeon notes, therefore clarity of the data could not be controlled. Post-operative medication use was available, although medication data available was not sufficient to allow complete assessment of the medication used to treat CRS before surgery (e.g., oral or topical cortical steroids and antibiotics).

Patients were not matched with regards to follow-up time, although the use of survival analysis and Cox-Proportional Hazards modelling allowed patients to be censored and accordingly included in the analysis when they were no longer followed-up.

### 4.4 Conclusion

This study has demonstrated the use of Chitogel following ESS procedures has a positive impact on long-term patient outcomes with flow-on effects to health system efficiency and cost-saving. Over the 2-year post-operative period, patients that were not treated with Chitogel were 2.39 times more likely to require revision surgery than patients that were treated with Chitogel. The upfront cost of using Chitogel in surgery was substantially offset by savings associated with reduced probability for revision surgery, improvements to health system efficiency, and indirect savings through improved patient health and reduced workplace absences. These findings suggest that use of Chitogel after ESS may improve patient outcomes and economic outcomes.

## Data Availability

The raw data supporting the conclusions of this article will be made available by the authors, without undue reservation.
