# Supplementary figures and images for: Chitogel improves long-term health economic outcomes following endoscopic sinus surgery in severe chronic rhinosinusitis patients
Source: Front Health Serv. 2024 Feb 28;4:1196499. doi: 10.3389/frhs.2024.1196499 (PMC10933009; doi:10.3389/frhs.2024.1196499)

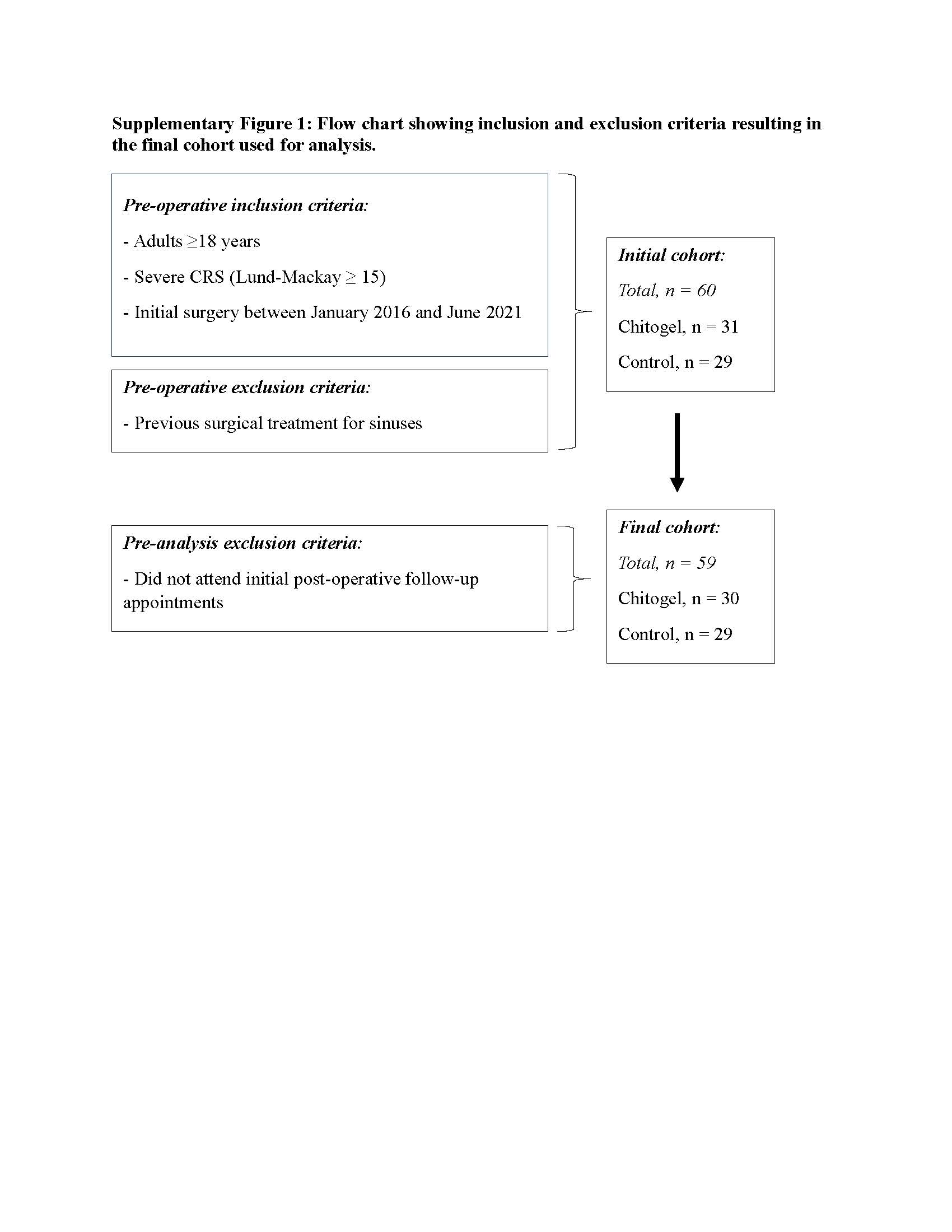

Supplement: Supplementary file 1 [file Image1.jpeg]
